# Drug-Metabolizing Activity, Protein and Gene Expression of UDP-Glucuronosyltransferases Are Significantly Altered in Hepatocellular Carcinoma Patients

**DOI:** 10.1371/journal.pone.0127524

**Published:** 2015-05-26

**Authors:** Linlin Lu, Juan Zhou, Jian Shi, Xiao-juan Peng, Xiao-xiao Qi, Ying Wang, Fang-yuan Li, Fu-Yuan Zhou, Liang Liu, Zhong-Qiu Liu

**Affiliations:** 1 International Institute for Translational Chinese Medicine, Guangzhou University of Chinese Medicine, Guangzhou, Guangdong, 510006, PR China; 2 Department of Pharmaceutics, School of Pharmaceutical Sciences, Southern Medical University, Guangzhou, Guangdong, 510515, PR China; 3 Department of Infectious Diseases, Nanfang Hospital, Southern Medical University, Guangzhou, Guangdong, China; 4 State Key Laboratory of Quality Research in Chinese Medicine, Macau University of Science and Technology, Macau (SAR), 999078, PR China; The Ohio State University, UNITED STATES

## Abstract

UDP-glucuronosyltransferases (UGTs), the most important enzymes in body detoxification and homeostasis maintaining, govern the glucuronidation reaction of various endogenous and environmental carcinogens. The metabolic function of UGTs can be severely influenced by hepatocellular carcinoma (HCC), the fifth prevalent and third malignant cancer worldwide. Particularly in China, HBV-positive HCC account for approximately 80% of HCC patients. But rare papers addressed the alteration on the metabolism of UGTs specific substrates, translational and transcriptional activity of UGTs in HBV-positive HCC patients. In present study, we choose the main UGT isoforms, UGT1As, UGT1A1, UGT1A9, UGT1A4 and UGT2B7, to determine the alterations of metabolic activity, protein and gene expression of UGTs in HBV-positive HCC. The corresponding specific substrates such as genistein, SN-38, tamoxifen, propofol and zidovudine were utilized respectively in UGTs metabolic activity determination. Furthermore, the plausible mechanism responsible for UGTs alterations was addressed by analyzing the protein and gene expressions in tumor and the adjacent normal tissues in HBV-positive HCC. The results revealed that in the tumor human liver microsomes (HLMs), either V_max_ (maximum reaction rate, R_max_ for UGT1A1) or the clearance rates (V_max_/K_m_, Clint) of UGT1A, UGT1A1, UGT1A4, UGT1A9 and UGT2B7 were significant lower than those of in the adjacent normal HLMs. Subsequently, the relative protein and gene expressions of these isoforms were notably decreased in most of tumor tissues comparing with the adjacent normal tissues. More interestingly, in tumor tissues, the metabolic activity reduction ratio of each UGT isoform was closely related to its protein reduction ratio, indicating that decreasing protein level would contribute to the reduced metabolic function of UGTs in HBV-positive HCC. In summary, our study firstly determined the alteration of UGT function in HBV-positive HCC patients, which would provide an important insight for toxicity or efficacy determination of chemotherapeutic drugs, and even bring a new strategy for clinical regimen in the health cares for the relative patients.

## Introduction

UDP glucuronosyltransferases (UGTs), the predominant phase II enzymes, play critical roles in homeostasis-maintain and detoxification. By participating in biotransformation of various endogenous compounds such as bilirubin, sex steroids and thyroid hormones, UGTs significantly contribute to constitutive cellular metabolic pathways [1]. On the other hand, UGTs are the cellular defense system by inactivation or increasing solubility of approximatively 35% of xenobiotics and current drugs, which could facilitate the excretion of lipophilic molecules from the body [2].

UGTs, consisting of four superfamilies (UGT1, UGT2, UGT3, and UGT8) which share 50 amino acids conserved UGT sequence, are mostly expressed in liver, kidney, colon, small intestine, esophagus and stomach [1]. Two major families, UGT1As and UGT2Bs, mainly abundantly expressed in the liver, were significantly contributing to biotransformation and metabolism of drug and endogenous compounds. The stable function and polymorphisms of hepatic UGT1As and UGT2Bs have notable impacts on body homeostasis. For instance, the high level of unconjugated hyperbilirubinemia in fetus, which was ascribe to the complete absence of UGT1A1 activity, could results in Crigler-Najjar’s syndrome type I, brain damage, and even death [3]. Moreover, because some exogenous carcinogens (including polycyclic hydrocarbons and heterocyclic amines) are subjected to conjugation reaction conducted by UGT1As and UGT2Bs [4], the basis protein, activity and polymorphism of individual these UGTs could determine predisposition towards cancer risk. Also, UGT1A9 exerts cancer prevention via inactivating benzo(α)pyrene (BaP) by glucuronidation [5]; UGT1A7*3 was regard as the sensitive polymorphism for carcinogens exposure due to its low catalytic activity [6]; metabolism of Diethylstilbestrol (DES) [7] and 4-(methylnitrosamino)-1-(3-pyridyl) -1-butanone (NNK) [8] conduct by UGT2B7 could influence chemical-induced carcinogenesis.

Furthermore, hepatic UGT1As and UGT2Bs were the vital enzymes involved in metabolizing wide variety of key anticancer agents, such as irinotecan, 7-ethyl-10-hydroxycamptothecin (SN-38) [9–11], sorafenib [12] and epirubicin [13,14], which were metabolized by UGT1A1, UGT1A9 and UGT2B7, respectively. Therefore, the altered metabolic activities or transcriptional alterations of these enzyme proteins in the liver could result in pharmacological and toxicological implications of these first line therapeutic drugs, which ultimately leading to the unexpected side effects or less efficacy in clinical cancer treatment. It was reported that the protein expression and metabolic activity of UGTs were closely related to the progression of liver diseases such as Crigler-Najjar type I [3], cirrhosis [15], nonalcoholic steatohepatitis (NASH) [16], and most importantly influence by hepatocellular carcinoma (HCC) [17,18], the fifth most frequent neoplastic disease in human with a mortality rate of 94% [12,19]. Particularly in China, approximately 75% to 80% of HCC patients have a hepatitis B virus (HBV)-infection background for 15 to 20 years. HBV carriers have a 10–100 fold risk of developing HCC compared with uninfected people [20,21]. However, whether the gene transcription, protein expression and function of UGTs are all impaired in the HBV-positive HCC still remains unclear. Specially, the precise information on the alteration of UGT1As and UGT2B7 in HBV-positive HCC tumor and the adjacent normal liver tissues is currently unavailable.

Therefore, in the present study, tumor and the corresponding adjacent normal liver tissues were collected from five individual HBV-positive HCC patients and were subjected to microsome preparations. The metabolic activities of major isoforms in UGT1A, UGT1A1, UGT1A9, UGT1A4 and UGT2B7 in HBV-positive HCC tumor human liver microsomes (HLMs) and the adjacent normal HLMs were investigated, respectively, by utilizing the specific substrate of each UGT such as SN-38 [11,22,23], propofol [24], genistein [25], tamoxifen and zidovudine. The protein expressions and transcriptional levels of UGT1A, UGT1A1, UGT1A9, UGT1A4 and UGT2B7 were also detected to consequently explore the relationship between metabolic activities and enzyme protein expression. Understanding the alteration of UGT1As and UGT2B7 in HBV-positive HCC will help elucidate the mechanism of response or resistance to chemotherapy in human liver cancer. The information of the interaction among the basis protein level, transcriptional activation and metabolic function will also help in the rational design of selective anti-tumor drugs and drug modulation, thereby resulting in improved therapeutic outcomes.

## Materials and Methods

### Chemicals and Reagents

SN-38, genistein, propofol, tamoxifen and zidovudine were purchased from Yingxuan Chempharm Co., Ltd. (Shanghai, China), Mansite Pharmaceutical Co., Ltd. (Chengdu, China), Tokyo Chemical industry Co., Ltd. (Tokyo, Japan), Maya Reagent Co., Ltd (Zhejiang, China), and Melone Pharmaceutical Co., Ltd (Dalian, China), respectively. SN-38G, propofol glucuronide, tamoxifen N-β-D-glucuronide and zidovudine O-glucuronide were purchased from Toronto Research Chemicals (North York, Canada). Uridine diphosphate glucuronic acid (UDPGA), alamethicin, D-saccharic-1, 4-lactone monohydrate, MgCl_2_, and β-glucuronidase were purchased from Sigma-Aldrich (St. Louis, MO, USA). The primary antibodies of anti-UGT1A (sc-271268), anti-UGT1A4 (sc-27427), anti-UGT2B7 (ab126269), anti-UGT1A1 (ab458411), and anti-UGT1A9 (H00054600-A01) were obtained from Santa Cruz Biotechnology, Inc. (CA, USA), Abcam (Cambridge, MA), and Abnova (Taipei, Taiwan), respectively. All other chemicals and reagents used were of the highest commercially available grade.

### Tissue samples and preparation of human liver microsomes

HBV-positive HCC tumor and the adjacent normal liver tissues of individual patients (*n* = 5, Table 1), who had undergone surgery for HCC resection, were obtained from the Affiliated Nanfang Hospital of Southern Medical University, Guangzhou, China. All procedures of tissue collection and in vitro xenobiotic metabolism studies were approved by the Nanfang Hospital Research Ethics Committee. Tissue samples were kept in ice-cold saline immediately after surgery, and subjected to microsome preparation as previous within 30 min [26]. Briefly, after washed with ice-cold buffer (8 mM KH_2_PO_4_, 5.6 mM Na_2_HPO_4_, 1.5 mM EDTA, 1 M DTT, and 0.28 mM phenylmethylsulfonyl fluoride), the tissues were then minced and homogenized in 50 mM phosphate buffer (containing 250 mM sucrose and 1 mM EDTA, pH 7.4). The homogenate was subjected to differential ultracentrifugation, 12,000×*g* for 15 min at 4°C, and then 110,000×*g* for 1 h at 4°C. The microsomal pellets were resuspended in 250 mM sucrose and immediately stored (10 mg to 50 mg protein/mL) at -80°C. The total protein concentrations were measured according to Bradford method, in which bovine serum albumin was used as the standard. The pooled tumor HLMs and the adjacent normal HLMs were subjected to kinetics comparison among SN-38G (UGT1A1 activity), propofol glucuronide (UGT1A9 activity), genistein glucuronide (UGT1As activity), and tamoxifen N-β-D-glucuronide (UGT1A4 activity) and zidovudine O-glucuronide (UGT2B7 activity). Besides that, five tumor and the adjacent normal human liver microsomes (HLMs) were subjected to determine the individual metabolism velocity towards each specific substrate.

**Table 1:** Clinical characteristics of human liver tissue donors.

Tissue number	Gender	Age (years)	HBV infection	Histology^a^ and Histological grade	TNM^b^	AFP^c^ (μg/L)	Volume^d^(cm^3^)
**P1-A06161**	Male	43	+	HCC(IIIa)	T_3_N_0_M_0_	>1000	58.9
**P2-A07271**	Male	43	+	HCC(IIIa)	T_3_N_0_M_0_	>1000	56.45
**P3-A10141**	Male	40	+	HCC(IIIa)	T_3_N_0_M_0_	>1000	111.4
**P4-A11101**	Male	42	+	HCC(IV)	T_3_N_0_M_0_	181.6	364.8
**P5-A12221**	Male	55	+	HCC(IV)	T_3_N_0_M_0_	>1000	58.55

^a^ Histological; HCC, Hepatocellular carcinoma

^b^ TNM classification

^c^ AFP, Alpha-fetoprotein

^d^ Tumor volume was determined by two-dimensional Volume*(cm^3^) using the formula: length*width^2^/2, length and width of tumor were diagnosed by US or CT

### UPLC analysis

An Agilent 1290 Infinity LC system were used, and Zorbax Eclipse Plus C18 column (1.8 μm, 2.1 mm × 50 mm), B Eclipse Plus C18 column (1.8 μm, 2.1 mm × 50 mm), and Zorbax RRHD SB-18 column (1.8 μm, 3 mm × 100 mm) were obtained from Agilent Technologies (CA, USA). The chromatography parameters were showed in S1 Table.

### Activity determination of UGT1A, UGT1A1, UGT1A9, UGT1A4 and UGT2B7

SN-38, genistein, propofol, tamoxifen and zidovudine were used as corresponding substrates for UGT1A1, UGT1As, UGT1A9, UGT1A4 and UGT2B7, respectively. The glucuronidation activities were determined in tumor HLMs and the adjacent normal HLMs collected from five HBV-positive HCC patients, and the pooled commercial HLMs were used as a reference. Same amounts of microsomes protein (0.265mg/ml) from different tissues were subject to determine the enzymatic activities of individual UGTs. Briefly, different combinations of microsomal protein amounts and incubation times were tested in preliminary studies to control the extent of metabolism to < 30% of the parent compound. A typical incubation mixture (final volume = 200 μL) containing 50 mM potassium phosphate buffer (for genistein and propofol) or 100 mM Tris-HCl buffer (for SN-38, zidovudine and tamoxifen) at pH 7.4. Human liver microsomes (final concentration = 0.0265 mg to 0.265 mg), MgCl_2_ (0.88 mM), saccharolactone (4.4 mM), alamethicin (0.022 mg/mL), different concentrations of substrates (genistein, 0.3125 μM to 50 μM; SN-38, 0.78 μM to 50 μM; propofol, 15 μM to 1200 μM; tamoxifen, 10μM to 100 μM; zidovudine, 250μM to 3500 μM), and UDPGA (3.5 mM, added last) were used. The mixture was incubated at 37°C for a predetermined period of time (typically within 90 min). As the internal standard, 50 μM testosterone was used in the reactions of genistein, SN-38 and propofol, while 22.4 μM molatrexed dihydrochloride and 0.5 μM chlorzoxazone were used in the reactions of tamoxifen and zidovudine, respectively. After the protein was removed by centrifugation at 13,500 rpm for 20 min, 15 μL to 20 μL of the supernatant was subjected to UPLC.

### Glucuronidation Kinetics

The rates of genistein, SN-38, propofol, tamoxifen and zidovudine metabolism in tumor HLMs and the adjacent normal HLMs were indicated as the amounts of metabolites formed per minute per milligram of protein. Kinetic parameters were then obtained according to the profile of Eadie-Hofstee plots described as before [27]. Same amounts of microsomes protein (0.265mg/ml) from different tissues were subject to determine the enzymatic activities of individual UGTs. If the Eadie-Hofstee plot is linear, the glucuronide formation rates (*V*) fit the standard Michaelis-Menten equation:

$$V=\frac{V_{\text{max }}\times \;C}{K_{m\;}+\;C}$$ (1)

where *K*_m_ is the Michaelis-Menten constant and *V*_max_ is the maximum rate of glucuronide formation. The data from atypical profiles were used in Eqs (2) or (3) on ADAPT II software when Eadie-Hofstee plots revealed characteristic profiles of atypical kinetics (autoactivation and biphasic kinetics).

$$\text{Reaction rate}=\frac{V_{\text{max }1}\times \;C}{K_{m\;1}+\;C}\;+\;\frac{V_{\text{max }2}\times \;C}{K_{m2}+\;C}$$ (2)

where *V*_max1_ and *V*_max2_ are the maximum enzyme velocities of the high- and low-affinity phases, respectively; *K*_m1_ is the substrate concentration that reaches 50% of *V*_max1_ for the high-affinity phase; and K_m2_ is the substrate concentration that reaches 50% of *V*_max2_ for the low-affinity phase.

$$\text{Reaction rate}=\frac{V_{\text{max }1}}{1\;+\;(K_{m\;1}/\text{ C})\;+\;(C/K_{\text{si}})}$$ (3)

where *V*_max1_ is the maximum enzyme activity of UGT isoform, *C* is the substrate concentration, *K*_m1_ is the substrate concentration that reaches 50% of *V*_max_ for one UGT isoform, and *K*_si_ is the substrate inhibition constant.

### Western blot

The protein expression levels of UGTs in tumor and the adjacent normal HLMs were determined by Western blot. Microsomes (50 μg) were separated on a 10% SDS polyacrylamide gel electrophoresis, and transferred onto PVDF membranes. The membranes were blocked with 5% non-fat milk in TBST (1h to 3h) and then incubated with primary antibodies of UGT1A (1:1000), UGT1A1 (1:500), UGT1A9 (1:500), UGT1A4 (1:1000) and UGT2B7 (1:1000) at 4°C overnight. After washed with TBST thrice, the membranes were incubated for 1 h with 1:2000 secondary antibodies (HRP-goat anti-mouse and HRP-goat anti-rabbit antibodies). The density of bands was determined by chemiluminescence. Calnexin was used as an internal control for protein loading.

### Total RNA extraction and reverse transcription from human Liver tissues

Total RNA was extracted from tumor and the adjacent normal liver tissues by using a PureLink RNA mini kit (Ambion, life technologies) and reverse-transcribed using a Primescript RT reagent kit (TaKaRa, Shiga, Japan) according to the manufacturer’s instructions. The mRNA expression levels were analyzed using ABI Prism 7500 with SYBR Premix Ex Taq II (TaKaRa, Shiga, Japan). All of the RT-PCR experiments were performed in duplicate. The levels of gene transcripts were quantified using the 2^-ΔΔ^CT method [28] and normalized to GAPDH (internal control). The primers used are shown as follows: UGT1A1, 5'-ATGCTGTGGAGTCCCAGGGC-3', 5'-CCATTGATCCCAAAGAGAAAACC-3'; UGT1A9, 5'-GAGGAACATTTATTATGCCACCG-3', 5'-CCATTGATCCCAAAGAGA AAACC-3'; UGT1A: 5'-ACTGGAACCCGACCATCGAATCTT-3', 5'-CACCAAACAAGGGCATCATCACCA-3'; UGT1A4: 5'-GAACAATGTATCTTTGGCCC-3', 5'-ACCACATCAAAGGAAGTAGCA-3'; UGT2B7: 5'-TGGTGTGGGCAGCAGAATACA-3', 5'-AGAGCGGATGAGTTGTTGGGAT-3'; GAPDH, 5'-GGCCTCCAAGGAGTAAGACC-3', 5'-AGGGGAGATTCAGTGTGGTG-3'.

### Statistical Analyses

One-way ANOVA with or without Tukey-Kramer multiple comparison (post hoc) tests were used to evaluate statistical differences. Student’s *t*-test was used as an additional test. Differences were considered significant when *p*-values were < 0.05. The correlation analysis was performed by Pearson correlation and Spearman’s rho in SPSS 19.0.

## Results

### Pathological characteristics of HBV-positive HCC tumor specimens

Given the gender difference in HBV-positive HCC incidence [20], five male HCC patients who had been infected with HBV for over 5 years, were enrolled in this study. These patients had a median age of 44.6 years, and as expected, AFP (α-fetoprotein) level of each patient were consistently high in plasmid. Tumor nodules were macroscopically classified by the presence or absence of a tumor capsule, intratumoral septum, and intratumoral necrosis. Based on the TNM classification parameters listed in the Cancer Staging Manual (AJCC, 7th edition, 2012), all of the liver tumor tissues were histologically confirmed as HCC (from stage IIIa to stage IV) by hematoxylin and eosin staining assay. And volumes of tumor tissues, which were defined as HBV-positive HCC tumor tissues in the following descriptions, were ranged from 56.45 cm^3^ to 364.8 cm^3^ (Table 1).

### The kinetics of each specific substrate glucuronidation in pooled commercial, tumor and adjacent normal HLMs

**Fig 1:**
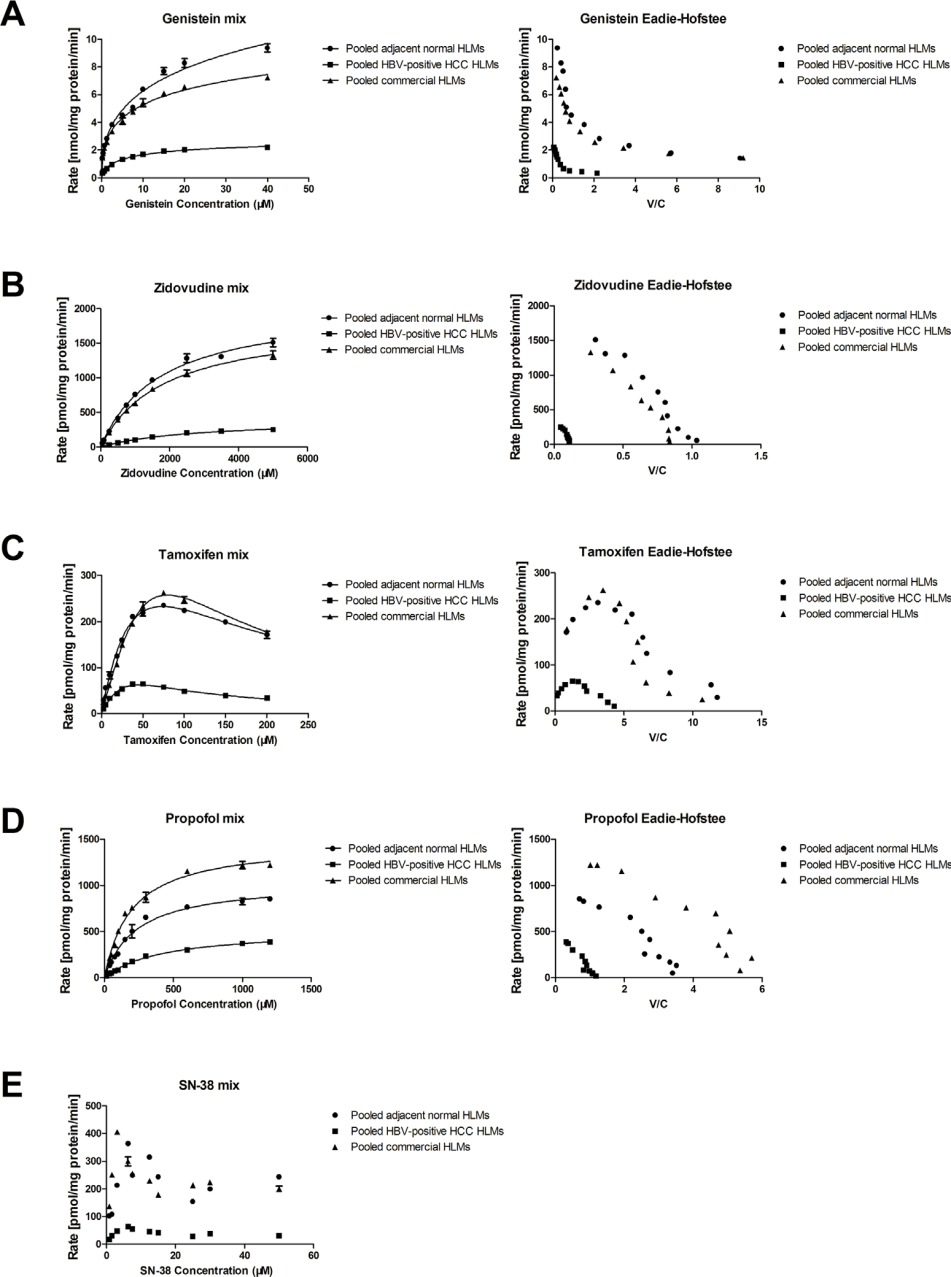
The kinetics of each specific substrate glucuronidation in pooled commercial, adjacent normal and HBV-positive HCC human liver microsomes. (A) Metabolism kinetics of genistein in commercial, adjacent normaland HBV-positive HCC HLMs (n = 5). (B) Metabolism kinetics of zidovudine in commercial, adjacent normal and HBV-positive HCC HLMs (n = 5). (C) Metabolism kinetics of tamoxifen in commercial, adjacent normal and HBV-positive HCCHLMs (n = 5). (D) Metabolism kinetics of propofol in commercial, adjacent normal and HBV-positive HCC HLMs (n = 5). (E) Metabolism kinetics of SN-38 in commercial, adjacent normal and HBV-positive HCC HLMs (n = 5). Each data point represents the average of three determinations. Representative data from three independent experiments are shown as the mean ± S.D.

To determine alterations of metabolic activity in the different microsomes, we utilized genistein, SN-38, tamoxifen, propofol and zidovudine as corresponding specific substrate for UGT1A, UGT1A1, UGT1A4, UGT1A9 and UGT2B7, respectively. The Eadie-Hofstee plot of genistein *Ο*-glucuronide indicated that the reaction kinetics of genistein was in accordance with the biphasic enzyme kinetics model (Fig 1A), while zidovudine and propofol glucuronidation were accordance with Michaelis-Menten kinetics model (Fig 1B and 1D). And based on the Eadie-Hofstee plots, the kinetics of tamoxifen metabolism to tamoxifen N-β-D-glucuronide was analyzed and determined as substration inhibition kinetics (Fig 1C). However, due to the fact that the SN-38 glucuronidation rate curves did not fit in any known model, the kinetic parameters of SN-38G were not obtained, but the maximum reaction rate (Rmax) of SN-38G was determined (Fig 1E).

The metabolic activity of UGT1As in pooled adjacent normal HLMs was consistent with that of in pooled commercial HLMs in terms of V_max_ (V_max1_: 6.71 ± 0.16 vs. 10.11 ± 0.72 nmol/mg protein/min; V_max2_: 1.83 ± 0.15 vs. 2.13 ± 0.47 nmol/mg protein/min). Similarly, no differences were found between pooled commercial HLMs and pooled adjacent normal HLMs in the metabolic activities of UGT1A1 (*R*_max_: 405.59 ± 4.19 vs. 364.39 ± 4.43 pmol/mg protein/min), UGT1A4 (V_max_: 743.20 ± 148.60 vs. 672.60 ± 123.30 pmol/mg protein/min), UGT1A9 (V_max_: 1466.75 ± 43.91 vs. 1043.69 ± 40.85 pmol/mg protein/min) and UGT2B7 (V_max_: 1830.83 ± 59.27 vs. 2050.89 ± 50.58 pmol/mg protein/min) (Table 2). However, in pooled tumor HLMs, the metabolic activities of UGT1As and UGT2B7 were significantly decreasing, such as UGT1A (V_max1_: 2.21 ± 0.05 nmol/mg protein/min; V_max2_: 0.04 ±0.33 nmol/mg protein/min), UGT1A1 (63.04 ± 3.60 pmol/mg protein/min), UGT1A4 (226.80 ± 63.37 pmol/mg protein/min), UGT1A9 (524.03 ± 16.62 pmol/mg protein/min) and UGT2B7 (405.48 ± 19.23 pmol/mg protein/min) (Table 2). Moreover, the clearance (*V*_max_/*K*_m_, Clint) of each substrate was impaired significantly in pooled tumor HLMs compared to the pooled adjacent normal HLMs. For example, the clearance rate of zidovudine was decreased by 87.93% in pooled tumor HLMs, and that of propofol was reduced by 71.97%.

**Table 2:** The kinetic parameters of genistein, zidovudine, tamoxifen, propofol and SN-38 glucuronidation in pooled commercial human liver microsomes (HLMs), pooled adjacent normal HLMs and pooled tumor HLMs.

	Kinetic Parameters	Pooled commercial HLMs	Pooled adjacent normal HLMs	Pooled tumor HLMs
**Genistein**	K_m1_ (μmol)	8.93 ± 0.93	14.08 ± 3.76	5.75 ± 0.53
	V_max1_ (nmol/mg protein/min)	6.71 ± 0.16	10.11 ± 0.72	2.21 ± 0.05
	V_max1_/K_m1_(μl/mg protein/min)	0.75	0.72	0.40
	K_m2_ (μmol)	0.06 ± 0.03	0.10 ± 0.09	0.04 ± 0.33
	V_max2_ (nmol/mg protein/min)	1.83 ± 0.15	2.13 ± 0.47	0.29 ± 0.05
**Zidovudine**	K_m_ (μmol)	1844.61 ± 43.85	1759.43 ± 64.37	2885.37 ± 28.65
	V_max_ (pmol/mg protein/min)	1830.83 ± 59.27	2050.89 ± 50.58	405.48 ± 19.23
	V_max_/K_m_(μl/mg protein/min)	0.99	1.16	0.14
	R^2^	0.99	0.99	0.99
	AIC	64.72	99.18	49.95
**Tamoxifen**	K_m_ (μmol)	89.08 ± 23.37	69.77 ± 17.37	60.06 ± 21.89
	V_max_ (pmol/mg protein/min)	743.20 ± 148.60	672.60 ± 123.30	226.80 ± 63.37
	V_max_/K_m_(μl/mg protein/min)	8.34	9.64	3.78
	R^2^	0.99	0.99	0.98
	AIC	61.15	72.05	49.31
**Propofol**	K_m_ (μmol)	196.42 ± 16.58	233.97 ± 24.72	420.77 ± 30.84
	V_max_ (pmol/mg protein/min)	1466.75 ± 43.91	1043.69 ± 40.85	524.03 ± 16.62
	V_max_/K_m_(μl/mg protein/min)	7.47	4.46	1.25
	R^2^	0.99	0.99	0.99
	AIC	109.71	105.83	76.24
**SN-38**	R_max_ (pmol/mg protein/min)	405.59 ± 4.19	364.39 ± 4.43	63.04 ± 3.60

### The metabolism velocity of genistein, protein and mRNA expression level of UGT1As in tumor and adjacent normal tissues

**Fig 2:**
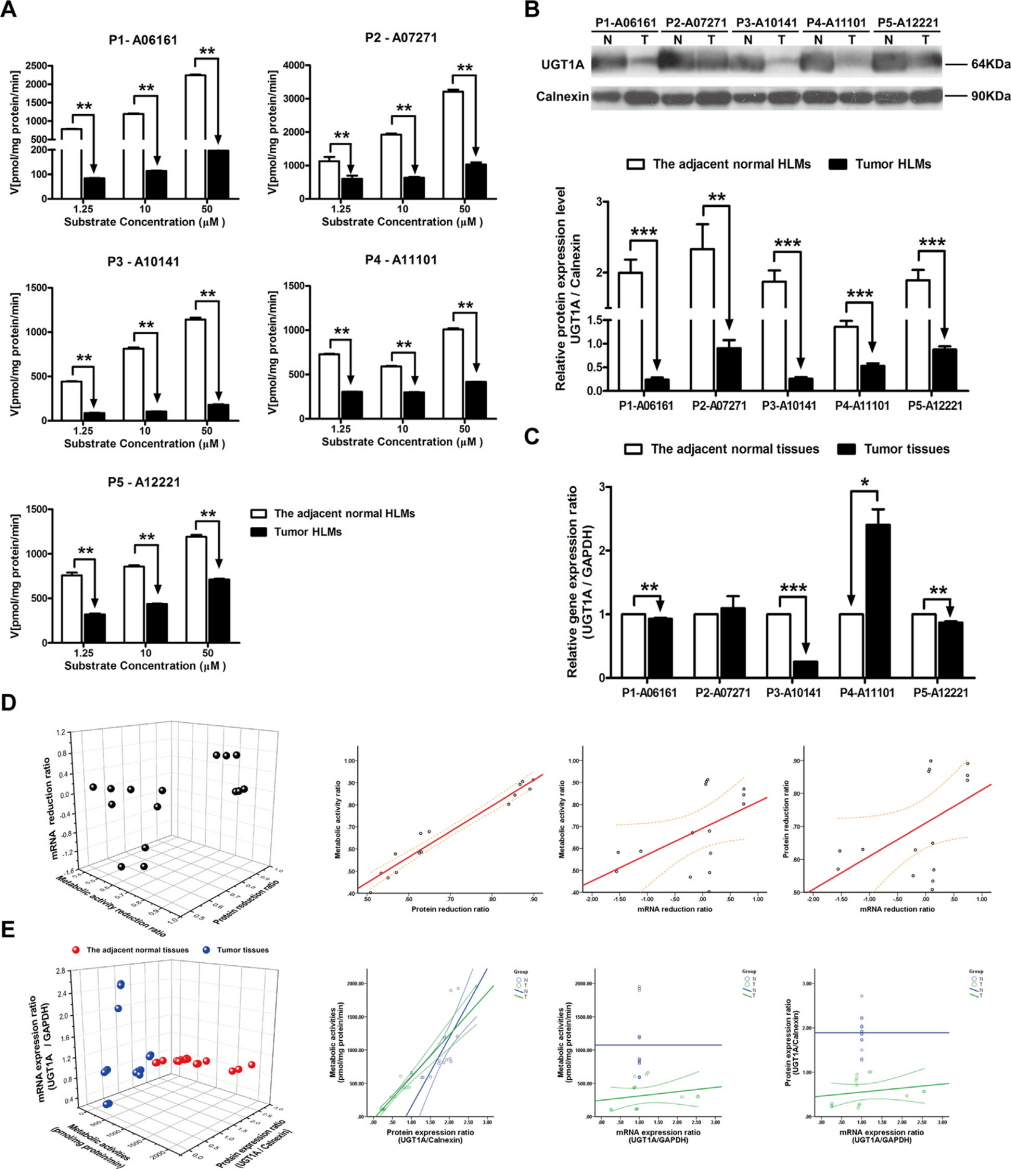
The metabolic activities, protein expression ratio and mRNA expression ratio of UGT1A in adjacent normal and HBV-positive HCC tissues. (A) Metabolism kinetics of genistein in commercial, adjacent normal and HBV-positive HCC HLMs (n = 5). (B) Protein expression ratio of UGT1A in adjacent normal (N) and HBV-positive HCC (T) HLMs. Calnexin was applied for protein loading normalization. (C) mRNA expression ratio of UGT1A in adjacent normal (N) and HBV-positive HCC (T) tissues. GAPDH was used for normalization. Each data point represents the average of three determinations. Representative data from three independent experiments are shown as the mean ± S.D, *, P<0.05; **, P<0.01; ***, P<0.001. (D) The pairwise correlation of UGT1A between metabolic activity reduction ratio, protein expression reduction ratio and mRNA expression reduction ratio. (E) The pairwise correlation of UGT1A between metabolic activities, protein expression ratio and mRNA expression ratio in the adjacent normal tissues or HBV-positive HCC tissues. Bivariate correlation analysis was used to analyze the relationship of all indexes (95% confidence interval).

By using three concentrations of specific substrate for UGT1As, the metabolic velocities of genistein in five individual tumor and the corresponding adjacent normal HLMs were analyzed. Compared to the adjacent normal HLMs, the metabolism velocity of genistein was consistently decreased in all of five tumor HLMs, particularly in P1-06161 with the average reduction of 90.33% (*P* < 0.01, Fig 2A) in three substrate concentration reactions (from 1.25 μM to 50 μM). And similar significant decreasing of UGT1As protein expression level was also found in five tumor HLMs comparing with the adjacent normal HLMs, the most reduction was also observed in P1-06161 (87.95%, *P* < 0.001, Fig 2B). Whereas notable individual differences of UGT1As gene expression level were detected in tumor tissues and the corresponding adjacent normal tissues. As shown in Fig 2C, comparing with the corresponding adjacent normal tissues, the mRNA expression level of UGT1As was dramatically decreased in tumor tissues of P1-06161 (*P* < 0.01), P3-10141 (*P* < 0.001) and P5-12221 (*P* < 0.01), while oppositely increased in P4-11101 (*P* < 0.05), and meanwhile, no difference was found in P2-07271. In order to investigate the predominant factor influenced the metabolic activity of each enzyme isoform, the reduction ratio of protein and mRNA expression level between tumor and the adjacent normal tissues were analyzed and subjected to correlation analysis. And correlation among the accurate detected values of metabolic activities, protein and gene expression levels of in individual five paired tissues were also analyzed. The metabolic activity ratio was closely related to protein reduction ratio (R^2^ = 0.979, *P* < 0.001) than to mRNA reduction ratio (R^2^ = 0.275, *P* < 0.321) in tumor and adjacent normal tissues. Furthermore, the correlation of protein reduction ratio and mRNA reduction ratio is very poor (R^2^ = 0.264, *P* < 0.341) (Fig 2D, Table 3). Meanwhile, significant correlations were observed between the metabolic activity and protein expression ratio either in tumor tissues (R^2^ = 0.929, *P* < 0.001) or in the adjacent normal tissues (R^2^ = 0.882, *P* < 0.001) (Fig 2E, Table 4).

**Table 3:** The pairwise correlation of UGT1As and UGT2B7 among metabolic activity reduction ratio, protein expression reduction ratio and mRNA expression reduction ratio.

	Metabolism-Protein	Metabolism-mRNA	Protein-mRNA
**UGT1A**	**R** ^**2**^ **= 0.979** ^b^, ***P*<0.001**	R^2^ = 0.275^b^, *P* = 0.321	R^2^ = 0.264^b^, *P* = 0.341
**UGT1A1**	**R** ^**2**^ **= 0.725** ^b^,***P* = 0.002**	R^2^ = 0.354^b^, *P* = 0.196	R^2^ = 0.218^b^, *P* = 0.435
**UGT1A4**	**R** ^**2**^ **= 0.869** ^**a**^, ***P*<0.001**	R^2^ = 0.057^b^, *P* = 0.840	R^2^ = 0.257^b^, *P* = 0.355
**UGT1A9**	**R** ^**2**^ **= 0.971** ^b^,***P* = 0.001**	R^2^ = 0.050^b^, *P* = 0.860	R^2^ = 0.164^b^, *P* = 0.558
**UGT2B7**	**R** ^**2**^ **= 0.996** ^b^,***P*<0.001**	R^2^ = 0.164^b^, *P* = 0.558	R^2^ = 0.214^b^, *P* = 0.443

^a^Pearson Correlation

^b^Spearman's rho

Bold represents a significant difference.

**Table 4:** The pairwise correlation of UGT1Asand UGT2B7 amongmetabolic activities, protein expression ratio and mRNA expression ratio in the adjacent normal tissues or tumor tissues.

	The adjacent normal tissue			Tumor tissue		
	Metabolism-Protein	Metabolism-mRNA	Protein-mRNA	Metabolism-Protein	Metabolism-mRNA	Protein-mRNA
**UGT1A**	**R** ^**2**^ **= 0.882** ^b^, ***P*<0.001**			**R** ^**2**^ **= 0.929** ^b^, ***P*<0.001**	R^2^ = 0.429^b^, *P* = 0.111	R^2^ = 0.286^b^, *P* = 0.302
**UGT1A1**	R^2^ = 0.488^a^, *P* = 0.065			**R** ^**2**^ **= 0.700** ^b^, ***P* = 0.004**	**R** ^**2**^ **= 0.593** ^b^,***P* = 0.020**	R^2^ = 0.330^a^, *P* = 0.229
**UGT1A4**	R^2^ = -0.008^a^, *P* = 0.976			**R** ^**2**^ **= 0.614** ^b^, ***P* = 0.015**	R^2^ = -0.093^b^, *P* = 0.742	R^2^ = 0.293^b^, *P* = 0.289
**UGT1A9**	R^2^ = -0.164^b^, *P* = 0.558			**R** ^**2**^ **= 0.811** ^b^, ***P*<0.001**	R^2^ = -0.486^b^, *P* = 0.066	R^2^ = 0.061^b^, *P* = 0.830
**UGT2B7**	R^2^ = 0.093^a^, *P* = 0.742			**R** ^**2**^ **= 0.828** ^b^, ***P*<0.001**	R^2^ = -0.018^b^, *P* = 0.950	R^2^ = 0.245^b^, *P* = 0.379

^a^Pearson Correlation

^b^Spearman's rho

Bold represents a significant difference.

### The metabolism velocities of SN-38, protein and mRNA expression levels of UGT1A1 in tumor and the adjacent normal tissues

**Fig 3:**
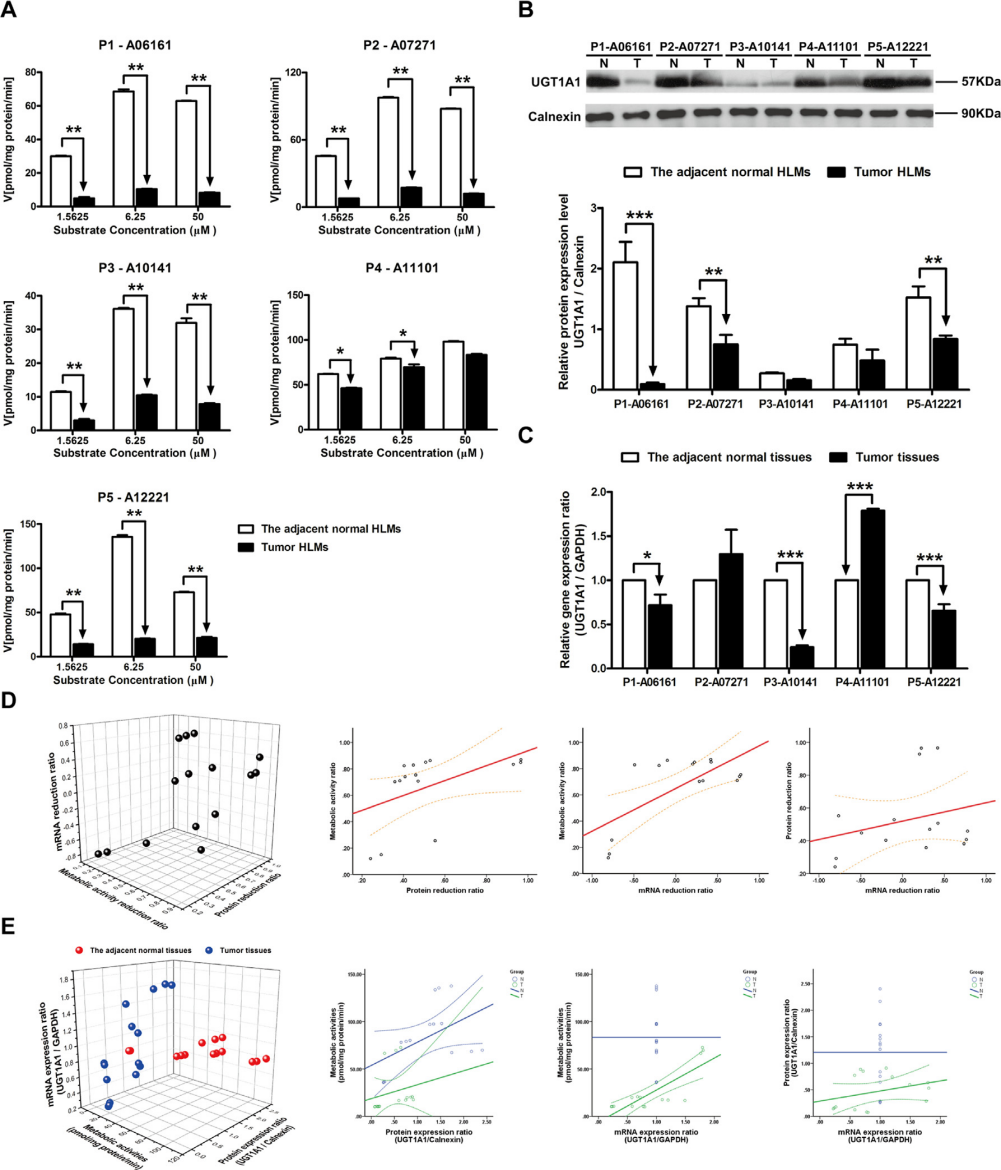
The metabolic activities, protein expression ratio and mRNA expression ratio of UGT1A1 in adjacent normal and HBV-positive HCC tissues. (A) Metabolism kinetics of SN-38 in commercial, adjacent normal and HBV-positive HCC HLMs (n = 5). (B) Protein expression ratio of UGT1A1 in adjacent normal (N) and HBV-positive HCC (T) HLMs. Calnexin was applied for protein loading normalization. (C) mRNA expression ratio of UGT1A1 in adjacent normal (N) and HBV-positive HCC (T) tissues. GAPDH was used for normalization. Each data point represents the average of three determinations. Representative data from three independent experiments are shown as the mean ± S.D, *, P<0.05; **, P<0.01; ***, P<0.001. (D) The pairwise correlation of UGT1A1 between metabolic activity reduction ratio, protein expression reduction ratio and mRNA expression reduction ratio. (E) The pairwise correlation of UGT1A1 between metabolic activities, protein expression ratio and mRNA expression ratio in the adjacent normal tissues or HBV-positive HCC tissues. Bivariate correlation analysis was used to analyze the relationship of all indexes (95% confidence interval).

When investigating the metabolic velocities of SN-38, with addition of low (1.5625 μM) and medium (6.25 μM) concentration of substrate, the metabolic rates of SN-38 in all of tumor HLMs were notably decreased comparing with the adjacent normal HLMs (*P* < 0.05, Fig 3A). However, in P4-11101, no significant difference in the metabolic activity was detected between the tumor and the adjacent normal HLMs when 50 μM of SN-38 was used. Comparing with the adjacent normal HLMs, the basis protein expression level of UGT1A1 was also decreased in three of five tumor HLMs (*P* < 0.01, Fig 3B). And subsequently, the mRNA expression level of UGT1A1 was also significantly down-regulated in three of five HBV-positive HCC tumor tissues (*P* < 0.05), while individual differences were also observed in P2-07271 and P4-A11101 (Fig 3C). The metabolic activity ratio was closely related to the protein reduction ratio between tumor tissues and the adjacent normal tissues (R^2^ = 0.725, *P* < 0.01) (Fig 3D, Table 3), and the accurate metabolic activities were significantly related to the protein and gene expression ratios of UGT1A1 (R^2^ = 0.700, *P* < 0.01; R^2^ = 0.593, *P* < 0.05) only in tumor tissues (Fig 3E, Table 4).

### The metabolism velocity of tamoxifen, protein and mRNA expression level of UGT1A4 in tumor and adjacent normal tissues

**Fig 4:**
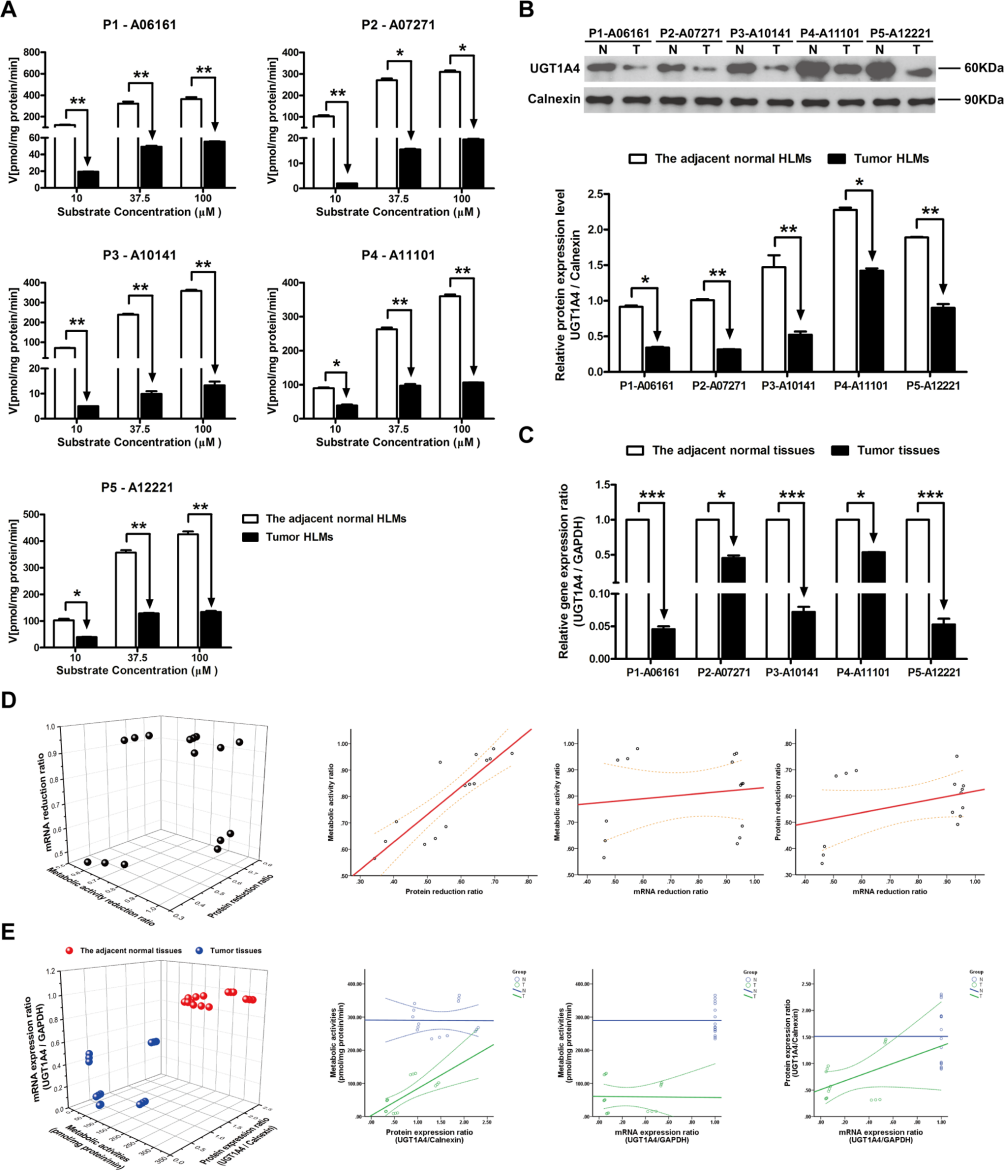
The metabolic activities, protein expression ratio and mRNA expression ratio of UGT1A4 in adjacent normal and HBV-positive HCC tissues. (A) Metabolism kinetics of tamoxifen in commercial, adjacent normal and HBV-positive HCC HLMs (n = 5). (B) Protein expression ratio of UGT1A4 in adjacent normal (N) and HBV-positive HCC (T) HLMs. Calnexin was applied for protein loading normalization. (C) mRNA expression ratio of UGT1A4 in adjacent normal (N) and HBV-positive HCC (T) tissues. GAPDH was used for normalization. Each data point represents the average of three determinations. Representative data from three independent experiments are shown as the mean ± S.D, *, P<0.05; **, P<0.01; ***, P<0.001. (D) The pairwise correlation of UGT1A4 between metabolic activity reduction ratio, protein expression reduction ratio and mRNA expression reduction ratio. (E) The pairwise correlation of UGT1A4 between metabolic activities, protein expression ratio and mRNA expression ratio in the adjacent normal tissues or HBV-positive HCC tissues. Bivariate correlation analysis was used to analyze the relationship of all indexes (95% confidence interval).

Comparing with the adjacent normal HLMs, the metabolic rates of tamoxifen in all of tumor HLMs were notably decreased regardless the concentration of substrate (from 10 μM to 100 μM) (*P* < 0.05, Fig 4A). Similarly, significant decreasing protein and mRNA levels of UGT1A4 were also observed in tumor subsequently (Fig 4B and 4C). The most reduction of protein level was found in P1-07271 (68.66%, *P* < 0.01), while the gene expression level was mostly down-regulated in P2-06161 (95.42%, *P* < 0.001). Furthermore, the correlation between the metabolic activity ratio and protein reduction ratio was tight (R^2^ = 0.869, *P* < 0.001) (Fig 4D, Table 3). While the metabolic activities of UGT1A4 was only significantly related to protein expression ratio in HBV-positive HCC tumor tissues (R^2^ = 0.614, *P* < 0.05) (Fig 4E, Table 4).

### The metabolism velocity of propofol, protein and mRNA expression level of UGT1A9 in tumor and adjacent normal tissues

**Fig 5:**
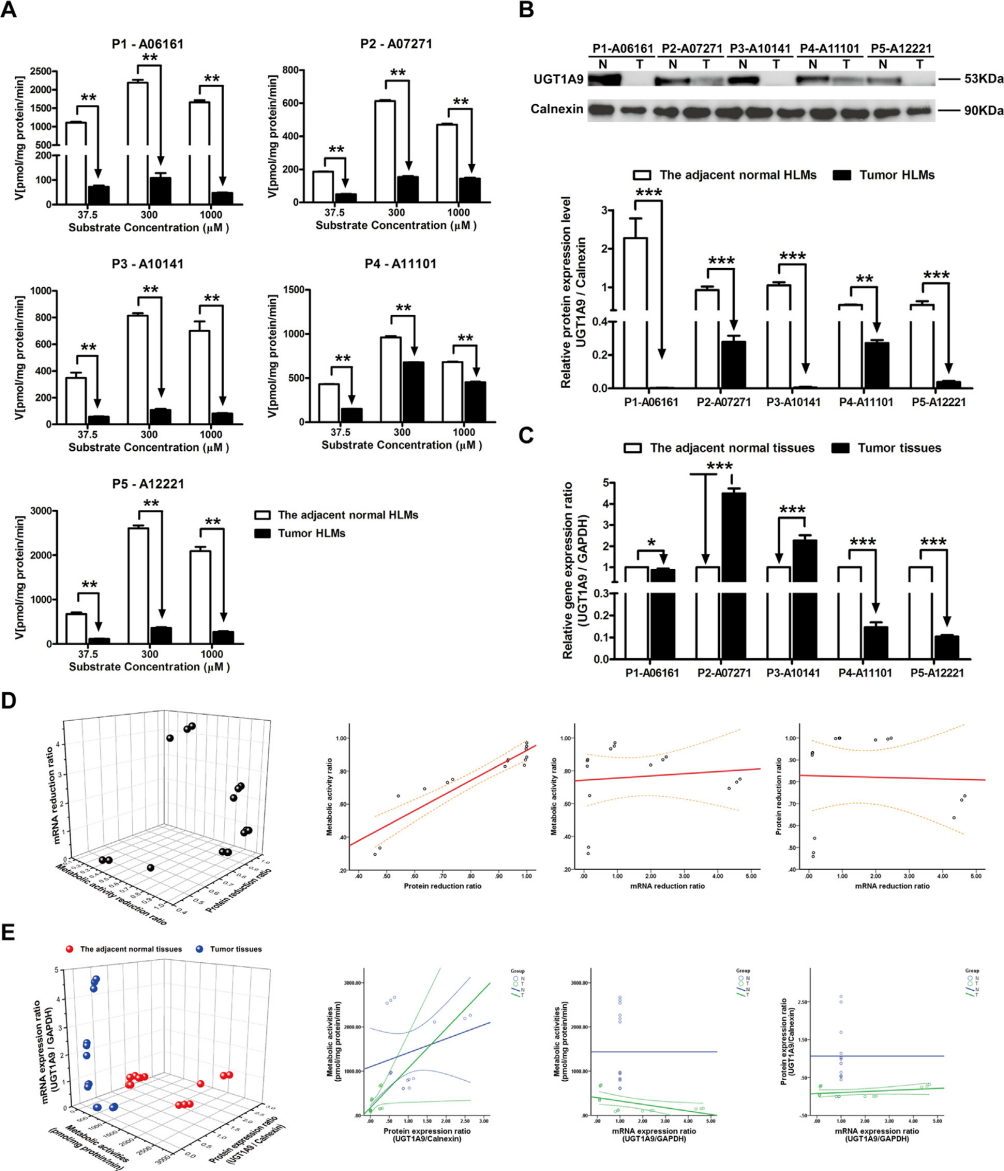
The metabolic activities, protein expression ratio and mRNA expression ratio of UGT1A9 in adjacent normal and HBV-positive HCC tissues. (A) Metabolism kinetics of propofol in commercial, adjacent normal and HBV-positive HCC HLMs (n = 5). (B) Protein expression ratio of UGT1A9 in adjacent normal (N) and HBV-positive HCC (T) HLMs. Calnexin was applied for protein loading normalization. (C) mRNA expression ratio of UGT1A9 in adjacent normal (N) and HBV-positive HCC (T) tissues. GAPDH was used for normalization. Each data point represents the average of three determinations. Representative data from three independent experiments are shown as the mean ± S.D, *, P<0.05; **, P<0.01; ***, P<0.001. (D) The pairwise correlation of UGT1A9 between metabolic activity reduction ratio, protein expression reduction ratio and mRNA expression reduction ratio. (E) The pairwise correlation of UGT1A9 between metabolic activities, protein expression ratio and mRNA expression ratio in the adjacent normal tissues or HBV-positive HCC tissues. Bivariate correlation analysis was used to analyze the relationship of all indexes (95% confidence interval).

By utilizing increasing concentration of propofol (from 37.5 μM to 1000 μM), the significant decreasing metabolic activities of UGT1A9 were found in five individual tumor and the adjacent normal HLMs (*P* < 0.01). Most average reduction of metabolic activity was observed in P1-06161 (95.22%, *P* < 0.01), while the least reduction of that was detected in P4-11101 (42.63%, *P* < 0.01) (Fig 5A). Correspondingly, the relative protein expression level of UGT1A9 was dramatically decreased in tumor HLMs compared to the adjacent normal HLMs, especially in P1-06161 with the reduction of 99.91% (Fig 5B). However, opposite differences of gene expression ratio between tumor and the adjacent normal tissues were also present, specifically, the gene expression level of UGT1A9 was notably increased in P2-07271 (452.63%, *P* < 0.001), while that of was significantly decreased in P5-12221 (90.86%, *P* < 0.001) (Fig 5C). Other than that, the tight correlation between the metabolic activity ratio and protein reduction ratio was found (R^2^ = 0.971, *P* < 0.001) (Fig 5D, Table 3). Whereas UGT1A9 metabolic activities was only significantly related to protein expression ratio in HBV-positive HCC tumor tissues (R^2^ = 0.811, *P* < 0.001) (Fig 5E, Table 4).

### The metabolism velocity of zidovudine, protein and mRNA expression level of UGT2B7 in tumor and adjacent normal tissues

**Fig 6:**
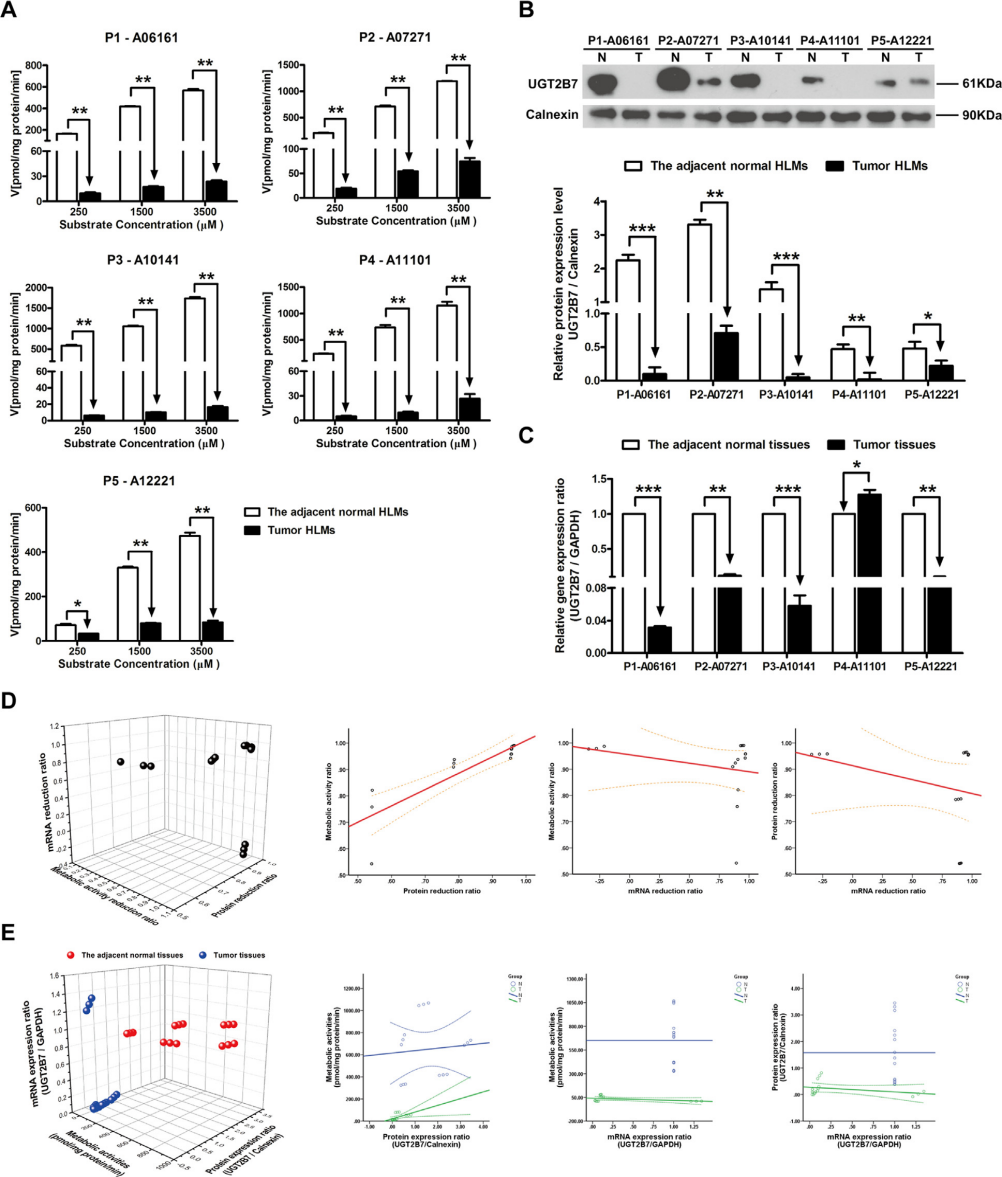
The metabolic activities, protein expression ratio and mRNA expression ratio of UGT2B7 in adjacent normal and HBV-positive HCC tissues. (A) Metabolism kinetics of zidovudine in commercial, adjacent normal and HBV-positive HCC HLMs (n = 5). (B) Protein expression ratio of UGT2B7 in adjacent normal (N) and HBV-positive HCC (T) HLMs. Calnexin was applied for protein loading normalization. (C) mRNA expression ratio of UGT2B7 in adjacent normal (N) and HBV-positive HCC (T) tissues. GAPDH was used for normalization. Each data point represents the average of three determinations. Representative data from three independent experiments are shown as the mean ± S.D, *, P<0.05; **, P<0.01; ***, P<0.001. (D) The pairwise correlation of UGT2B7 between metabolic activity reduction ratio, protein expression reduction ratio and mRNA expression reduction ratio. (E) The pairwise correlation of UGT2B7 between metabolic activities, protein expression ratio and mRNA expression ratio in the adjacent normal tissues or HBV-positive HCC tissues. Bivariate correlation analysis was used to analyze the relationship of all indexes (95% confidence interval).

Compared to the adjacent normal HLMs, the metabolic velocity of zidovudine in all of tumor HLMs were notably decreased with the most reduction in P3-10141 (99.01%, *P* < 0.01, Fig 6A). Subsequently, the relative protein and mRNA expression levels of UGT2B7 were significantly decreased in tumor tissues (Fig 6B and 6C). The most reduction of protein level was found in P3-10141 (96.38%, *P* < 0.001), while the gene expression level was mostly down-regulated in P1-06161 (97.23%, *P* < 0.001). Moreover, the tight correlation was found between the metabolic activity ratio and protein reduction ratio (R^2^ = 0.996, *P* < 0.001) (Fig 6D, Table 3), and between the UGT1A4 metabolic activities of individual HLMs and the relative protein expression level in HBV-positive HCC tumor tissues (R^2^ = 0.828, *P* < 0.001) (Fig 6E, Table 4).

## Discussion

Glucuronidation reaction, which conducted by UGTs metabolic system, represents a central pathway for cellular defense, and determines the effectiveness of delivery and administration of therapeutic agents [29]. UGTs, which conjugate the sugar moiety to the multiple sites of lipophilic molecules in xenobiotics and environmental compounds, exert a latent capacity for evolutionary innovation due to its glucose entail viability [30]. Thus, investigating the metabolic function of UGTs and exploring the mechanism responsible for is necessary and important for understanding the biological system of human body. However, due to the overlapping substrate specificities, high level of sequence identity, lack of protein structure and active site definition and limitation of *in vitro*-*in vivo* extrapolation, to properly predict the exact isoform of UGTs participated in certain drug glucuronidation still remains highly challenging [1]. Therefore, the metabolism of UGTs should be determined individually before predicting their function and behavior *in vivo*. In this study, five human liver tissue specimens resected from patients were collected in our study and subjected to metabolic activity analysis by using specific substrates for respective isoform of UGT1As and UGT2B7 (Table 1).

Although some published studies revealed that the gene expression levels of UGTs were significantly down-regulated in nonalcoholic steatohepatitis (NASH) [16] and pre-malignant adenomatous hyperplasia of the liver [18], the altered transcriptional and translational activities of UGTs were still under controversial in the progression of liver diseases [31]. Furthermore, to our knowledge, the metabolic activities and catalytic function of UGTs were rarely addressed in HCC, more especially in HBV-positive HCC. Given the increasing incidence and mortality of HCC with the high HBV-infection background in China, we systematically analyzed the metabolic function, protein and gene expression levels of UGT1A, UGT1A1, UGT1A9, UGT1A4 and UGT2B7 in HBV-positive HCC tumor and the adjacent normal tissues. By utilizing certain specific substrates for UGTs, we initially demonstrated that catalytic activities of UGT1A, UGT1A1, UGT1A4, UGT1A9 and UGT2B7 were significantly decreased in the tumor tissues in HBV-positive HCC patients (Figs 1 and 2A–6A). And complementary to the fact that the gene and protein expression levels of UGTs were not altered in the benign tumors [18], we also found that the metabolic activities of UGT1As and UGT2B7 in the adjacent normal HLMs were consistent with that of in the commercial (absolute normal) HLMs (Fig 1, Table 2).

In our following study, notable reductions in the protein and gene expression levels of UGT1As and UGT2B7 were subsequently observed in most of tumor tissues compared to that in adjacent normal tissues (Figs 2C and 2D–6C and 6D). Meanwhile, individual differences, such as contrary tendency of gene and protein expression level were also present in tumor tissues. Several studies showed that the polymorphisms of each UGTs isoform, even combination of single nucleotide polymorphisms (SNPs) could be the potential factors contributing to these significant individual differences [29]. Moreover, as UGT1As and UGT2B7 are also the important enzymes for metabolizing daily essential nutrients such as flavonoids in fruits and polyphenols in green tea [27,32], thus, the consumption of these nutrients can also influenced the transcription and translation of UGTs in turn, which ultimately induced the individual differences. Most importantly, our study found that the correlation between the protein and mRNA expression level in human liver tissues is week (Table 4), which was consistent with studies revealed that translating mRNAs near the ribosome, other than total mRNA, strongly correlate to cellular protein synthesis [33,34]. Thus, in further study, translatomics would be a better strategy for exploring the interrelationship between protein and gene expression.

Some studies revealed that the enzymatically active isoforms of UGTs and the corresponding pseudoprotein (enzymatically inactive isoforms) could be distributed differently in tumor tissues and exert contrary tendency in protein expression. For example, the intense immunohistochemical expression of UGT1Ai2 (inactive isoform) was found in liver tumors while decreasing protein levels of UGT1Ai1 and UGT1Ai2 were observed in colon cancer specimens [35]. In order to determine predominate factor influenced the metabolic activities of UGTs, the interrelationship of metabolic activity, protein and gene expression level of each UGT isoforms was investigated. Comparing with other correlations, the metabolic activity ratio of UGT1As and UGT2B7, which means the reduction ratio of metabolic activity in tumor tissues, was closely related to the protein reduction ratio of these enzymes (Table 3). This tight correlation indicates that the decreasing protein expression level could be the prerequisite and plausible mechanism responsible for the reduced metabolic activities of UG1As, UGT1A1, UGT1A4, UGT1A9 and UGT2B7. And in turn, the significantly impaired function of UGT1As and UGT2B7 in tumor tissues implied their down-regulated translational activities, which were reported as the predisposing factors for hepatocarcinogenesis [1].

In summary, given the important roles of UGTs played in biotransformation and detoxification, the consistently decreased metabolic activities and protein expression level of UGT1As and UGT2B7 in our study will provide relevant information for the clinical regimen, forecasting the toxicity and efficacy of drugs in HBV-positive HCC patients, and for improving therapeutic outcome of first line anti-cancer agents such as tamoxifen and SN-38. On the other hand, the impaired function of UGTs may be served as a novel sensor for an early diagnosis indicator for hepatocarcinogenesis in prognosis of HCC infected by HBV. Furthermore, the individual differences of UGTs protein and gene expression levels in HCC patients also implied that monitoring the expressions and activities of UGTs in HCC tumors tissues of individual patients is necessary for further individualized treatment of HCC, or probably for drug designing.

## Supporting Information

S1 Table The chromatography parameters used in UPLC analysis. (DOC)
