# Supplementary material for: Drug-Metabolizing Activity, Protein and Gene Expression of UDP-Glucuronosyltransferases Are Significantly Altered in Hepatocellular Carcinoma Patients
Source: PLoS One. 2015 May 26;10(5):e0127524. doi: 10.1371/journal.pone.0127524 (PMC4444081; doi:10.1371/journal.pone.0127524)
Supplement: S1 Table — (DOC) [file pone.0127524.s001.doc]

**Supplement Table The chromatography parameters used in UPLC analysis**

| **Parameters**  **(LC-UV)** | **SN-38 and**  **its glucuronide** | **Genistein and**  **its glucuronide** | **Propofol and**  **its glucuronide** |
| --- | --- | --- | --- |
| Injection volume (μL) | 20 | 20 | 15 |
| Mobile phase (A/B)^a^ | 10 mM NH_4_Ac/ACN | 2.5 mM NH_4_Ac/ACN | Water/ACN |
| Gradient program  (%B (min)) | 10(0-0.8)→15(1.0)→50(3.6)  →90(3.9)→10(4.7-5) | 10(0-0.5)→50(3.1)→90(3.4)  →10(4.2)→10(4.5) | 10(0-0.6)→64(3.2)→85(3.9)  →10(4.5-5.5) |
| UV wavelength (nm) | 265 | 254 | 220 |
| Retention time (min) | 5 | 4.5 | 5.5 |
| Flow Rate (mL/min) | 0.4 | 0.5 | 0.35 |
| test linear range for the glucuronide (μM) | 0.076-5 | 0.076-50 | 0.1526-50 |
| **Parameters**  **(LC-UV)** | **Tamoxifen and**  **its glucuronide** | **Zidovudine and**  **its glucuronide** |  |
| Injection volume (μL) | 10 | 10 |  |
| Mobile phase (A/B)^a^ | 10 mM NH_4_Ac/ACN | 0.1% (v/v) Formic acid/ACN |  |
| Gradient program  (%B (min)) | 10(0-1.2)→30(3.6)→53(3.9)  →10(5.0) | 10(0-1.0)→60(3.0-3.4)  →10(4.0-5.5) |  |
| UV wavelength (nm) | 238 | 254 |  |
| Retention time (min) | 5 | 5.5 |  |
| Flow Rate (mL/min) | 0.4 | 0.3 |  |
| test linear range for the glucuronide (μM) | 10-300 | 250-3500 |  |

^a^pH (10 mM NH_4_Ac) = 4.0; pH (2.5 mM NH_4_Ac) = 3.0
